# Advance care planning after hospital discharge: qualitative analysis of facilitators and barriers from patient interviews

**DOI:** 10.1186/s12904-018-0379-0

**Published:** 2018-12-05

**Authors:** Vanessa Peck, Sabira Valiani, Peter Tanuseputro, Sunita Mulpuru, Kwadwo Kyeremanteng, Edward Fitzgibbon, Alan Forster, Daniel Kobewka

**Affiliations:** 1 Department of Medicine, The Ottawa Hospital, University of Ottawa, Ottawa, ON Canada; 2 Ottawa Hospital Research Institute, Ottawa, ON Canada

**Keywords:** Advance care planning, Conversation guide, Communication

## Abstract

**Background:**

Patients who engage in Advance Care Planning (ACP) are more likely to get care consistent with their values. We sought to determine the barriers and facilitators to ACP engagement after discharge from hospital.

**Methods:**

Prior to discharge from hospital eligible patients received a standardized conversation about prognosis and ACP. Each patient was given an ACP workbook and asked to complete it over the following four weeks. We included frail elderly patients with a high risk of death admitted to general internal medicine wards at a tertiary care academic teaching hospital. Four weeks after discharge we conducted semi-structured interviews with patients. Interviews were transcribed, coded and analysed with thematic analysis. Themes were categorized according to the theoretical domains framework.

**Results:**

We performed 17 interviews. All Theoretical Domain Framework components except for *Social/Professional Identity* and *Behavioral Regulation* were identified in our data. Poor knowledge about ACP and physician communication skills were barriers partially addressed by our intervention. Some patients found it difficult to discuss ACP during an acute illness. For others acute illness made ACP discussions more relevant. Uncertainty about future health motivated some participants to engage in ACP while others found that ACP discussions prevented them from living in the moment and stripped them of hope that better days were ahead.

**Conclusions:**

For some patients acute illness resulting in admission to hospital can be an opportunity to engage in ACP conversations but for others ACP discussions are antithetical to the goals of hospital care.

**Electronic supplementary material:**

The online version of this article (10.1186/s12904-018-0379-0) contains supplementary material, which is available to authorized users.

## Background

People with advanced life limiting disease near the end-of-life often want limitations on invasive life sustaining treatments and instead want care focused on comfort and quality of life [1, 2]. Contrary to this wish, treatment at the end of life often includes invasive life sustaining treatments such as mechanical ventilation and cardio pulmonary resuscitation [3, 4]. Advance Care Planning (ACP) is defined as a process that “enables individuals to define goals and preferences for future medical treatment and care, to discuss these goals and preferences with family and health-care providers, and to record and review these preferences if appropriate” [5]. Ongoing engagement in the ACP process is one step to ensuring that people receive care consistent with their goals. It is a particularly valuable process for those living with chronic, life limiting illnesses or the very elderly because they are more likely to require decisions about invasive and potentially unwanted life prolonging therapies [6].

ACP has many proven benefits. ACP increases satisfaction with care at the end of life and reduces anxiety and depression for surviving relatives [7]. Participation in ACP also results in lower health care costs [8]. Most importantly, engaging in ACP can help patients and their families understand what is important to them and communicate this to their health care team so they receive care consistent with their values and wishes [7, 9, 10].

Despite the many benefits of ACP, most older adults do not have a written document describing their wishes for medical care and less than half have discussed their wishes with a doctor [1, 11]. The lack of communication about end of life preferences means that patients’ health care choices aren’t being elicited or followed [12].

Previously identified patient barriers to ACP engagement include: perceived irrelevance because patients see themselves as too healthy, emotional barriers such as fear of death, and concerns about burdening family members with ACP discussions [13]. We hypothesized that admission to hospital will increase the relevance of ACP because patients will no longer feel they are too healthy [14]. There are numerous published studies on interventions to encourage ACP engagement that have reported varying levels of success [10]. Few of these trials investigate the modifiable barriers to ACP engagement and only one uses a behavioral framework to analyze their results and understand how their intervention facilitates or fails to facilitate ACP behaviors [13, 15]. We chose the Theoretical Domains Framework (TDF) for this study because it synthesizes many behavioral theories into domains that explain the psychological processes that regulate behavior [16, 17]. It is a useful framework for assessing implementation problems [18].

In Canada, a National Advance Care Planning task group has set goals for ACP engagement and created resources including conversation guides and ACP workbooks to help people engage in advance care planning [19, 20]. These resources have been designed by experts and promoted across the country but have not been evaluated extensively. The resources were not designed specifically for the hospital context but considering the opportunity to engage frail elderly people during hospitalization we educated physicians in the use of an ACP conversation guide and provided physicians with an ACP workbook to give to their patients prior to discharge. The objective of this study was to determine the facilitators and barriers to ACP engagement for frail elderly people who received this structured conversation and ACP workbook prior to discharge from hospital.

## Methods

### Approach and researcher characteristics

This study is reported according to the Standards for Reporting Qualitative Research (SRQR) recommendations [21] Additional file 1. We used a pragmatic paradigm with qualitative thematic analysis of patient semi-structured interviews. VP is a registered social worker with a background in qualitative analysis techniques. DK is a general internist. Neither were directly involved in the care of any patients recruited to the study. Both DK and VP participated in data collection and analysis. The study was approved by The Ottawa Health Science Network Research Ethics Board.

### Setting

The study took place on the General Internal Medicine Teaching Units (MTU) at a tertiary-care hospital in Ottawa, Ontario.

### Population

We included patients who were 85 years or older who were admitted for an acute medical problem or patients for whom the attending physician would not be surprised if they died in the next 6 months [22]. These criteria were chosen to pragmatically select patients with an increased risk of death for whom ACP is particularly important. Patients were excluded if they could not speak English or did not have capacity to make their own healthcare decisions as judged by their physician in hospital.

### Description of the intervention

All attending physicians and residents received training in the use of a conversation guide and an ACP Workbook. The training included the definition of ACP, the benefits of ACP activities for patients and a demonstration of the conversation guide with group discussion of barriers to using the guide. Both the conversation guide and the workbook have been developed by the Canadian Hospice and Palliative Care Association as a part of a National Framework and Implementation project [20].

Clinicians were asked to use the guide to discuss prognosis and explain the purpose of ACP with their patients once they had recovered from the acute phase of their illness, prior to discharge. At the end of this conversation, the physicians provided the patient with an ACP workbook (available from http://www.speakupontario.ca). The patient was asked to complete the book within one month of discharge.

### Recruitment

After a patient had received the intervention from their physician they were approached for consent to be contacted for a follow up interview after discharge. We continued recruiting patients until we had 3 consecutive interviews with no new codes identified.

### Interviews

Three weeks after discharge from hospital VP contacted consenting patients to coordinate semi-structured interviews. The interviews were completed in person, one-on-one at a location of the participant’s choosing. Each question explored the patients’ memory of the conversation in hospital and any contemplation or ACP actions (making of an advance care plan, discussing wishes with a loved one and designating a substitute decision maker) they had engaged in since discharge from hospital. The prompt after each question explored the facilitators and barriers for the patient to engage in the ACP related actions. Interview questions also explored how the 2 parts of our intervention (the conversation prior to discharge and ACP workbook) acted as facilitators or barriers to ACP engagement. The full interview guide is available in Additional file 2.

### Analysis

Interviews were audio-recorded, anonymized, transcribed verbatim and analyzed using thematic analysis. Two researchers (DK and VP) independently coded four transcripts then met to discuss codes and develop a coding strategy. The remainder of the interviews were coded by VP with 3 transcripts randomly reviewed by DK. Following coding, DK and VP met to categorize codes into emerging themes. We used an inductive approach where themes were mapped to the TDF to understand the barriers and facilitators of ACP activities [16]. The TDF was developed by a collaboration of behavioral scientists to group constructs from theories of behavior into a comprehensive list of the determinants of behavior. In order to understand how to improve our intervention in subsequent implementation work each TDF domain was further categorized into a component of the Behavior Change Wheel: Capability, Opportunity and Motivation (COM). The COM-B is a validated framework that links specific barriers and facilitators to interventions and policy types [18, 23].

## Results

### Participants

Patients were recruited between March and November 2016. A total of 32 patients gave informed consent to be contacted for interviews, 17 participated in interviews. The reasons for non-participation included: patients could not be reached after discharge, death, and withdrawal of consent. Fifteen patients were recruited because the attending physician answered “no” to the surprise question. Two patients were recruited because they were age 85 years or older admitted for an acute medical problem. Nine participants were male (53%) and 8 were female (47%). Ages of participants ranged from 57 to 89 with a mean age of 75. Interviews lasted between 20 to 120 min.

### Theoretical domain framework

Twelve out of 14 TDF domains were identified as barriers or facilitators to patients engaging in ACP activities post-discharge (Tables 1 and 2). We identified *Knowledge, Skills, Beliefs about Capabilities, Memory Attention and Decision Processes, Beliefs about Consequences, Social influences, Environmental Context and Resources, Emotion, Reinforcement, Intention and Goals* as domains that facilitated ACP engagement (Table 1). All of the same domains were identified as barriers except *Intention and Goals.* The additional domain of *Optimism* was identified as a barrier but not a facilitator of ACP engagement (Table 2). *Social/Professional Identity* and *Behavioral Regulation* were not identified in the data.

**Table 1:** Facilitators to Patient Engagement in ACP Post Hospital Discharge

Theoretical Domain Framework Category	Theme/Belief	Quote
Knowledge	Conversation outlined what’s involved in advance care planning.	“Planning requires more than just have done the power of attorney for personal care, that it requires having a frank conversation with your loved ones about what your wishes are and perhaps a little more specifically than what the power of attorney entails”. – 612 “It surprised me because when he talked about advance care planning I had no real idea what he was talking about but I presumed it was related to my illness at the time and what I could expect in the future in terms of additional care right and so when he came in and started explaining what this is about it was ah ok I haven’t spent a lot of time thinking about that and so subsequently I have as a result so I think that was the biggest benefit of the visit was that it spurred my mind in a direction that I had taken it before in any serious way”. - 618
	ACP workbook presented new information that made me reconsider my prior thinking about care preferences.	“The book gave a little bit of a hiccup in that that I guess I don’t know how to put in words the in-between stage that I see now… I just felt that if you had to be resuscitated that you wouldn’t have any quality of life but if there is a chance of having quality of life for some reason then I would be ok with it.” – 705
Skills	Quality of interaction with the physician.	“I think that’s critical to be able to communicate in a way that the patient can possibly understand what you’re talking about and to be really clear about the prognosis of somebodies illness because that’s probably sometimes hard information to give out but I think a doctor that withholds some of that or disguises it with terminology that is not well understood is not doing anybody any favors. So I think really beyond the technical capabilities communication skill is important and that to me has got to be the most important thing to be able to speak in a way that is understandable and clear”. –618 “The doctor in ICU last Saturday was excellent and he sat down. He knew that made a difference and had to go find a chair to bring over to sit down. In fact he was very touchy feely”. – 103
Beliefs about Capabilities	Talking about death is not difficult due to religious beliefs.	“I am a person of deep faith and so death is part of your faith and umm it’s just not something I have shied away from thinking about….I lost both of my parents in my twenties. We also adopted a child and he was a crib death two weeks after my father died so the death umm the knowledge and experience of death was early in my life”. – 973
Beliefs about Consequences	Death is unpredictable.	“I have to get my rear end in gear and get all this stuff in place and you know something could happen to me because I have been told by several doctors that the next time I could die”. – 701
Social Influences	Decisions about future care are relational.	“Being able to communicate my wishes to my family so that they understand them and have an opportunity to discuss them with me so that in the event of one of the circumstances coming up where a decision has to be made at least they have understood and appreciated in advance that’s what I would have wanted as opposed to being a surprise because in some cases they may totally disagree with the approach that I want to take. For example, you want to fight for every breath, live as long as possible no matter what vs I have no future therefore no unusual measures to prolong life. Well without a discussion that could be heartbreaking and so I would like to avoid that by being open about it that’s the only way”. – 618
Environmental Context and Resources	Discharge is an appropriate time to discuss ACP.	“I’m glad they talked to me right before discharge, I think it would have been a waste of time had it been a couple of weeks earlier. Ya it’s a time where you’re thinking about these things if you suffered a serious episode like I had and its things that are on your mind in any case so to be given some guidance at the time was very useful I think”. – 618
Emotion	Normalizing ACP conversations makes it easier to talk about and to accept.	“It’s again like I said the more people who know, the more you discuss it, the more you think about it, it becomes easier for everybody”. – 92
Reinforcement	The Speak Up workbook encouraged me to engage in ACP planning.	“The booklet helped to reflect on certain things and I think I got more information from the booklet as to what really he wanted me to think about…I think it helped pinpoint certain things that I had thought about and reinforced those things”. – 606 “This is a personal decision that requires a lot of thought and I think this brochure (Speak Up) along with the various websites that it refers to really allows you to have the information available to help that decision making process”. -618
	Hospital conversation encouraged me to think about and document ACP.	“I think I can appreciate that they were reminding me, us that there were things that had to been done…. A realization that there were a whole number of things that we had to talk about and plan for and I am still feeling like I may have a day or two left” -103 “Since I’ve gotten out of the hospital I have made a couple of appointments with my lawyer, ok I had a will made up 20 years ago and I wanted it updated, so I have two power of attorney’s now… So they will make the decision for example that there will be no resuscitation that’s all been settled and we talked about it”. -606
	Hospitalization a catalyst for advance care planning.	“I’ve thought about it before because I was in the hospital two years ago with heart failure and at that time I said you never know when somethings going to happen it could be tomorrow. And then I sort of let it go. Then I was in the hospital again this time and they had to take me off certain blood thinners and perform certain interventions and they said there’s always a certain risk that because were taking you off blood thinners you could have a blood clot or a stroke. Ok it’s my third time in the hospital for major things, I could have been a goner for all three of them so I said no I’m not going to wait for the fourth time so that’s what really…it was in the back of my head before but I would say ya ya it’s a ways away but this one it really hit home more than the others”. – 606
Intention	Relieving the decision-making burden on my loved ones.	“Basically at this stage of the game I am 84 I have lived a very good life, lots of tragedy in it but by and large a good life but it’s very important to me that my passing will be eased for my children. I don’t want any of this great upheaval, where are mothers papers, what did she want?” – 973
Goals	Planning for place of death.	“My husband died here in this house and I knew the last week what it was and he wouldn’t make it. The doctor came after his death would you believe that Dr. <Name> came at 11:30 at night and all my family was upstairs in the bedroom and he said do you know what a beautiful death this is? He said you go to the hospital and this isn’t the same thing. He said you have all this family around and that’s the way I want it… I want to die at home”. – 20
Memory, Attention and Decision Processes	Recollection of the in hospital conversation.	“What I remember, what he told me was that I had to think about my future and that I also have to think about myself…Yes it matters because I have to think of the future, I just can’t go on day by day and not worry about anything I have to you know”. - 701

**Table 2:** Barriers to Patient Engagement in ACP Post Hospital Discharge

TDF Domain	Theme/Belief	Quote
Knowledge	Lack of understanding about the seriousness of one’s illness.	“All he told me at the time which I didn’t know at the time was the severity of this GOUT. I did not realize how bad it was, I did not realize that I could be gone right now”. – 20 **“**I was very upset on Thursday when I came back of course because I did not understand all the ramifications of a pacemaker and a defibrillator and I did not understand that my heart was so sick”. – 973
	Widespread misunderstanding about what ACP is.	“I have done some arrangements made with the funeral parlors and this type of thing has been done since my husband passed away so I mean I have things kind of organized there and financial things with them and try and make it easier for them and I don’t know yet it’s hard to know if you only knew what was going to happen to you” – 617 “I am working on my funeral arrangements and I am also making a list of who gets what because I know after a death there can be arguments or not really arguments but people saying well I would like that so I would like to have as much as that settled and it makes it easier for those who are left behind. So I don’t want them to be bothered with funeral arrangements or deciding who gets what so that will be all. Now the advance care part, I haven’t really thought too much about it”. – 606
Skill	Physicians’ ability to effectively engage patients in conversations about prognosis, goals of care, and medical preferences at the end of life.	“I realize it’s difficult for them a lot of them it’s not easy to bring up the topic you’re getting stonewalled by patients they don’t want to hear it and the families making faces and saying what are you telling us and the whole bit but just the very basic communication skills get down to the patients level I mean how simple is that and don’t stand there like you’re ready to move on, and checking the blood pressure and ready to move just doesn’t cut it. And have something to back up what you’re saying because we all hear and learn differently whether it’s visual or oral”. – 103 “He was very snobbish. Just the way he came out with it not preparing us that he was going to talk about it. Here she thought my mother must be dying, no she’s not why are you talking to her like that?... He could have said we’re going to talk about if you’re prepared but instead he came out and whatever he said it was not taken how he probably meant it”. -302
Beliefs about Capabilities	Documenting ACPs is difficult due to the uncertainty around future health care needs.	“I don’t know what I’m going to need that’s the problem”. – 701 “It’s hard to put all these things together when you’re the person concerned and if you’d knew more as to what was going to happen to you and this type of thing you might be able to do more but you don’t know whether you’re going to become unconscious or whether you’re not and all this and it’s hard to imagine what it’s going to be like”. – 617
Beliefs about Consequences	Denial of seriousness of illness	“As a person with emphysema I know like it’s a dead end. Is that the right work to use? It’s a matter of time, some people last two months, some last ten years it all depends on the individual and how they look at, or the steps the take towards their own health. That’s what I think, my father died from emphysema and he had that since the age of 35 and he died at 56. So what do you do, how can you tell? I don’t think it’s a good thing for a doctor to say he has two months to live, I don’t think it’s the right thing to do”. – 91 “Well I think there has been certainly some very normal denial on your part and you have said more than once that you just really don’t want to be thinking about this”. – 103 “I don’t think nobody knows what the end is going to look like. Some people are lucky and they go in their sleep and some aren’t lucky they suffer longer. So I don’t know, I just….the way that I look at it now is it’s just a question of time until I recover and I don’t think it has a real impact right now in my life except for the time being I can’t do much”. – 601
Social Influences	Negative reactions about ACP discussions from family.	“My daughter got pretty mad about that, she’s not dying why do you want to talk to her about dying?” – 302 “We’ve started [talking about ACP] but they [children] don’t want to talk about it”. -103 “My daughters don’t want to hear about it… my youngest son is 54 and it bothers him very much and I said why? My life is an open book, why does that bother ya? He doesn’t like to see that. My oldest son is the one with the authority and I tried to talk to him about it but he sort of cleared that conversation. They don’t like to hear about it because I have always been outgoing and never sick except for the past two years and they find that hard to except”. -20 “<Name> he just says mummy I don’t want to talk about like when I was just out there as you know and he just I mentioned it briefly to him and I said <Name> were going to be talking to you about all this and he said mom I don’t want to talk about it and wrapped his arms me and started to cry you know”. – 701
Environmental Context and Resources	Timing of ACP discussions.	“It was just the timing I guess and its very I’m going to use the word awkward I don’t know if it’s an appropriate word but yes the timing was off, I don’t need to talk about this today, I want to focus on getting well”. - 612
	Patients’ perceived physicians were in a rush	“The people on the medicine unit were running out of time and had to get the task done quickly and did not have the time to take the time to discuss it in more detail”. - 103 “I found the doctors at the <Hospital name> really didn’t seem to have the time, they were quite hurried and under a time crunch”. – 618
	Hospital is not the appropriate place to have ACP discussions	“Sometimes in the hospital the ambiance is not very good cause I was in a room with two swearing people and they were screaming all night. The ambiance is not nice to start talking about this. In a private room maybe it’s different….I think it was not the place to have it right now it is the place to have. My place I am comfortable. I think it’s more cozy and more personal”. - 91 “First not in the room that I was in. They should have more private rooms you know. There’s the…they call it the T.V room and there’s never anybody in there. It would have been nice for them to have said let’s go and talk there or if I am alone in the bedroom then yes but not when there’s a sick person like that [beside me]”. 302
Optimism	Focus was on getting better not on possible end-of-life situations	“I was surprised by it as it was a very emotional time and so I guess I was trying to focus on whether <Name> would get well or not and so it was a little disturbing emotionally”. – 612 “I’m lying there in bed and I think I’m going to get better and I can imagine that 90% of people think they’re going to getbetter or their hoping their going to get better and then the doctor comes in and say’s you better do some thinking”. – 910
	Coping by living in the moment.	“I don’t want to talk about it to be honest with you. I want to live day by day and like you know what happens happens. One day I’m ok and the next day I’m not and that’s the way you have to take it. You have to take it day by day”. – 91
Emotion	ACP discussion brings up fears about loss of independence.	“I’ve been so independent I mean I brought up three kids on my own had no help, worked three jobs all my life, you know and now a lot of my independence has been taken away from me and that’s just killing me. That’s the part that I don’t like like when they discuss this part, like they did yesterday and I started to cry. You know because it’s just hurting me because there is just so much I can’t do anymore”. – 701
	It is hard to talk about ACP because I am scared of death.	“I just get very scared, very very scared of death”. – 701
Memory, Attention and Decision Processes	Very little recollection of the ACP in hospital conversation.	“Vaguely, I do. I was not in good shape. I was shaking, and had tears in my eyes” – 100 “Because I had everything in order I didn’t really pay that much attention, I guess because I thought I had everything in order”. – 705

#### Capabilities

##### Memory, attention and decision processes

Numerous participants did not remember the conversation about ACP while in hospital because they were too sick.

*“Vaguely, I do. I was not in good shape. I was shaking, and had tears in my eyes”*

Others remembered the conversation but were too sick to engage their physician in a discussion about ACP.

##### Knowledge

Knowledge was both a facilitator and barrier to ACP engagement. For some participants, the structured in-hospital conversation provided an opportunity to clarify their understanding of ACP and the practical steps involved. For these participants, the conversation incited further consideration of end-of-life planning and the workbook provided additional guidance to proceed with completing this process post-discharge.

*“It surprised me because when he talked about advance care planning I had no real idea what he was talking about but I presumed it was related to my illness at the time and what I could expect in the future in terms of additional care right and so when he came in and started explaining what this is about it was ah ok I haven’t spent a lot of time thinking about that…”*

However, knowledge also acted as a barrier for many participants in this study. First, many participants lacked understanding about their own health condition(s), prognosis, and what they were planning for. Second, for some patients there was generalized misunderstanding about ACP, the components of the planning process, and the role of advance care plans in relation to incapacity and consent. This lack of knowledge was exhibited by participants who often considered ACP to be synonymous with decisions about CPR, power of attorney documents, and/or living wills. In addition to this confusion, many participants believed that ACP was about finalizing funeral and financial arrangements for one’s death and felt that the in-hospital conversation was not necessary or helpful since they were already prepared for their own passing.

*“I am working on my funeral arrangements and I am also making a list of who gets what because I know after a death there can be arguments or not really arguments but people saying well I would like that…. Now the advance care part, I haven’t really thought too much about it.”*

From these accounts, it appears that some participants were more prepared for death rather than states of incapacity. This suggests that lack of awareness and understanding of ACP is a barrier to engagement in the process.

##### Skills

Many participants in this study indicated that the physicians’ interpersonal skills and communication abilities decreased their willingness to discuss difficult topics including prognosis and goals of care. Patients felt that it was necessary for physicians to be able to communicate in an honest and straightforward manner and that their approach towards discussing difficult topics needed to attend to their informational and emotional needs.

*“He was very snobbish. Just the way he came out with it not preparing us that he was going to talk about it. Here she thought my mother must be dying, no she’s not why are you talking to her like that?... He could have said we’re going to talk about if you’re prepared but instead he came out and whatever he said it was not taken how he probably meant it”.*

Empathetic and informative communication practices were described by patients as improving their understanding and recall of in-hospital ACP discussions and better prepared them to make medical, psychosocial, and spiritual decisions in relation to end-of-life planning.

*“The doctor in ICU last Saturday was excellent and he sat down. He knew that made a difference and had to go find a chair to bring over to sit down. In fact he was very touchy feely”.*

Physician communication skill (or lack of skill) was a strong facilitator and barrier to engaging patients in conversations about ACP.

#### Opportunity

##### Social influences

Patients saw ACP as a way to take the decision-making burden off of their loved ones. By making decisions for themselves patients were sparing their loved ones from “heart breaking” decisions and ensuring that their wishes would be respected. Social influences were also a barrier. Many patients expressed that their loved ones did not want to talk about ACP or about death in general.

##### Environmental context and resources

The timing of ACP discussions was a barrier. Many participants described not being in the right frame of mind to discuss ACP while in hospital and felt that it was untimely to have these discussions when they were focusing on getting better. For these participants, their focus was on their physical recovery and they were hesitant to engage in ACP discussions during their hospital stay.

*“It was just the timing I guess and its very I’m going to use the word awkward I don’t know if it’s an appropriate word but yes the timing was off, I don’t need to talk about this today, I want to focus on getting well”.*

Participants also raised concerns about the appropriateness of conducting ACP discussions in hospital rooms where there is often a lack of privacy and confidentiality. These participants believed that lack of privacy made communication challenging especially when discussions revolved around sensitive and emotionally charged topics like prognosis and goals of care. Finally, participants also perceived that their physicians lacked the time needed to engage in thorough ACP conversations.

The ACP workbook was a resource that allowed participants to engage in ACP at their own pace. Patients who were not well enough to recall and/or participate in ACP discussions while in hospital found that the workbook provided sufficient information for them to meaningfully engage in ACP post-discharge.

#### Motivation

##### Beliefs about capabilities

Some patients were comfortable talking about death because of past experiences. Others stated that they could not engage in ACP because they didn’t know what was going to happen in the future.

##### Beliefs about consequences

For some patients, the unpredictability of death was motivating because there was limited time to have ACP conversations.

*“I have to get my rear end in gear and get all this stuff in place and you know something could happen to me because I have been told by several doctors that the next time I could die”.*

Others denied that that death was likely and therefore thought that ACP was not a useful or relevant activity.

*“I don’t think nobody knows what the end is going to look like… So I don’t know, I just….the way that I look at it now is it’s just a question of time until I recover and I don’t think it (ACP) has a real impact right now in my life except for the time being I can’t do much”.*

##### Emotion & Optimism

The emotion and optimism domains were closely aligned in our data. Talking and thinking about ACP in the context of their own care evoked fears for participants. Some described difficulty coming to terms with the increased possibility of losing independence and autonomy while others expressed anxiety around their own death.

*“I just get very scared, very very scared of death”.*

Some participants did not want to think about or plan for the worst-case scenarios and described focusing their energy on living in the moment. Planning for the future was secondary to living now.

*“I don’t want to talk about it to be honest with you. I want to live day by day and like you know what happens happens. One day I’m ok and the next day I’m not and that’s the way you have to take it. You have to take it day by day”.*

Living in the moment for some participants re-ignited a sense of hope and optimism that perhaps better days were ahead and engaging in ACP discussion stripped them of this possibility.

*“I’m lying there in bed and I think I’m going to get better and I can imagine that 90% of people think they’re going to get better or they’re hoping they’re going to get better and then the doctor comes in and says you better do some thinking”. – 910*

Many participants had difficulty simultaneously balancing their hopes for the future alongside their need to plan for changes in their future health.

##### Reinforcement

Some patients described their hospitalization as an impetus for considering future planning. In these instances, the seriousness of their conditions coupled with their greater understanding of the risks associated with treatment options influenced their openness and readiness to talk about ACP.

*“I think I can appreciate that they were reminding me, that there were things that had to been done…. A realization that there were a whole number of things that we had to talk about and plan for…”*

For patients in the right frame of mind, ACP discussions in hospital reinforced the importance of end-of-life planning.

##### Goals & Intention

One patient was motivated to engage in ACP so they could die at home. Other patients had a goal of lessening the decision making burden on their family. These goals for their own death motivated them and gave them resolve in performing ACP activities.

*“…it’s very important to me that my passing will be eased for my children. I don’t want any of this great upheaval, where are mother’s papers, what did she want?” – 973*

## Discussion

We delivered a simple intervention to frail elderly patients in hospital that included a structured conversation about ACP, and a workbook to encourage ACP activities after discharge. We assessed barriers and facilitators to ACP engagement after discharge with semi-structured interviews and applied the theoretical domain framework to guide future implementation.

### Facilitators and barriers addressed by our intervention

Similar to other ACP interventions, some patients said they had improved knowledge about why and how to engage in ACP which improved their capability to perform ACP [24]. We found that the conversation in hospital and the workbook both reinforced the benefits of ACP and transmitted knowledge about how to perform it, even though lack of knowledge remained a barrier for some patients. Some patients were particularly receptive to information about ACP while in hospital, consistent with our hypothesis that admission to hospital was a catalyst that made ACP more relevant. Others did not think the timing was right to discuss ACP or had no recollection of receiving information about ACP. Even for those who did not feel that the timing was right, exposure to ACP materials may increase awareness and eventually lead to engagement in ACP activities, similar to health behaviors [25]. Introducing ACP prior to discharge will not be effective for all patients but it is an opportunity that should not be missed.

Some patients perceived that ACP was not relevant to them, a lack of motivation in the COM-B system. This has been identified as an important barrier to ACP engagement for patients in the community because they see themselves as healthy and do not see the relevance of planning for serious illness [13]. We found that even during acute illness some patients did not think ACP was important because their primary focus was on recovering from an acute illness. The phenomena of “not now” may originate from a desire to maintain hope by denying the possibility of death or prolonged illness. The need to maintain hope does not necessarily prevent patients from preparing for death if communication from the medical team focuses on achieving specific goals as opposed to focusing on what cannot be done [26]. Conversely, openly discussing the probability of survival has been associated with loss of meaning, and treatments that offer no benefit [27, 28]. Balancing hope with an understanding of illness trajectory can facilitate meaningful ACP but it is a tension for patients and healthcare professionals alike [29, 30].

The hospital environment was a barrier to ACP for some patients. Hospital rooms have little privacy and roommates are often disruptive. Finding quiet, private space to talk can be challenging but is important for quality communication [31, 32].

Physicians’ communication skills are known to be a critical factor in determining patient motivation and capability for a range of health problems [33, 34]. We anticipated that variation in communication skill would be an important determinant of success and therefore used a conversation guide to standardize the words used to describe ACP and its benefits. Despite using a guide to standardize conversations patients reported that the quality of communication impacted their openness to conversations about ACP. We did not measure fidelity of conversation guide use, so the effect of the conversation guide is uncertain. Our findings reinforce the importance of communication skills training although improving clinicians’ communication skill is difficult [35].

Similar to other ACP interventions we found that patients’ perception that physicians do not have time was a barrier to ACP discussions [36, 37]. Quality conversations require focused attention that is difficult when there is time pressure. Mindful communication training has been shown to improve physicians’ ability to listen attentively to patients. Although mindfulness will not increase the time available for conversations about ACP it may improve patients’ perception that physicians are in a rush [38].

### Facilitators and barriers not addressed by our intervention

We identified numerous barriers and facilitators to ACP engagement that were not addressed by our intervention. The *Emotion* domain with themes of fear of death and of losing autonomy, a barrier of motivation, have been found in previous research in this area [13]. Emotional barriers are difficult to overcome but simple communication techniques such as exploring and validating emotions or motivational interviewing have promise [39]. The *Social Influences* domain was an important barrier of opportunity in our data. Loved ones do not always want to engage in discussions about end-of-life and some people do not have family or friends with whom they feel comfortable sharing their wishes. Our intervention did not directly address this barrier. Future implementation could educate family members about the benefits of ACP for them and their loved one.

### Limitations

Our recruitment process likely selected for patients who were highly engaged in their own health care and therefore the barriers to ACP engagement in our study may not be generalizable to all hospitalized patients. Nevertheless, there is likely significant overlap between the barriers we identified and the barriers for less engaged patients. One limitation is that some patients did not even remember the conversation about ACP in hospital and therefore could not give specific input on how the intervention facilitated or acted as a barrier to their engagement in ACP. While this is a limitation it is also an interesting and important finding that deserves further exploration. Another limitation of our study is that we did not quantify the barriers. *Schickedanz* et al. performed a quantitative study of barriers to ACP engagement post discharge and found perceived irrelevance, which maps to the TDF domain of *Beliefs about Consequences,* and *Personal issues*, which maps to TDF domain *Emotions,* were the most prevalent barriers [13].

## Conclusions

Hospitalization can be a catalyst that increases the perceived relevance of ACP for some patients. Future iterations of this intervention need to target key barriers, including negative emotions, lack of privacy for sensitive conversations and physician communication skills while capitalizing on facilitators of ACP engagement.

## Additional files

Additional file 1: SRQR standards for Reporting Qualitative Research Checklist. Checklist for all elements that are suggested to be reported for qualitative research. (PDF 263 kb)

Additional file 2: Semi Structured Interview Guide. The interview guide used for semi-structured interviews of patients (DOCX 17 kb)

## References

[1] Heyland DK, Barwich D, Pichora D, Dodek P, Lamontagne F, You JJ, Tayler C, Porterfield P, Sinuff T, Simon J (2013). Failure to engage hospitalized elderly patients and their families in advance care planning. JAMA Intern Med.

[2] Heyland DK, Rocker GM, O'Callaghan CJ, Dodek PM, Cook DJ (2003). Dying in the ICU: perspectives of family members. Chest.

[3] Merchant RM, Yang L, Becker LB, Berg RA, Nadkarni V, Nichol G, Carr BG, Mitra N, Bradley SM, Abella BS (2011). Incidence of treated cardiac arrest in hospitalized patients in the United States. Crit Care Med.

[4] Kazaure HS, Roman SA, Sosa JA (2013). Epidemiology and outcomes of in-hospital cardiopulmonary resuscitation in the United States, 2000-2009. Resuscitation.

[5] Rietjens JAC, Sudore RL, Connolly M, van Delden JJ, Drickamer MA, Droger M, van der Heide A, Heyland DK, Houttekier D, Janssen DJA (2017). Definition and recommendations for advance care planning: an international consensus supported by the European Association for Palliative Care. Lancet Oncol.

[6] Milnes S, Orford NR, Berkeley L, et al. A prospective observational study of prevalence and outcomes of patients with gold standard framework criteria in a tertiary regional Australian hospital. BMJ Support Palliat Care. 2015. 10.1136/bmjspcare-2015-000864. 10.1136/bmjspcare-2015-000864 26391750

[7] Detering KM, Hancock AD, Reade MC, Silvester W (2010). The impact of advance care planning on end of life care in elderly patients: randomised controlled trial. Bmj.

[8] Zhang B, Wright AA, Huskamp HA, Nilsson ME, Maciejewski ML, Earle CC, Block SD, Maciejewski PK, Prigerson HG (2009). Health care costs in the last week of life: associations with end-of-life conversations. Arch Intern Med.

[9] Sudore RL, Boscardin J, Feuz MA, McMahan RD, Katen MT, Barnes DE (2017). Effect of the PREPARE website vs an easy-to-read advance directive on advance care planning documentation and engagement among veterans: a randomized clinical trial. JAMA Intern Med.

[10] Houben CHM, Spruit MA, Groenen MTJ, Wouters EFM, Janssen DJA (2014). Efficacy of advance care planning: a systematic review and meta-analysis. J Am Med Dir Assoc.

[11] Kirschner KL (2005). When written advance directives are not enough. Clin Geriatr Med.

[12] Heyland DK, Ilan R, Jiang X, You JJ, Dodek P (2016). The prevalence of medical error related to end-of-life communication in Canadian hospitals: results of a multicentre observational study. BMJ Qual Saf.

[13] Schickedanz AD, Schillinger D, Landefeld CS, Knight SJ, Williams BA, Sudore RL (2009). A clinical framework for improving the advance care planning process: start with patients' self-identified barriers. J Am Geriatr Soc.

[14] Reinke LF, Engelberg RA, Shannon SE, Wenrich MD, Vig EK, Back AL, Curtis JR (2008). Transitions regarding palliative and end-of-life care in severe chronic obstructive pulmonary disease or advanced cancer: themes identified by patients, families, and clinicians. J Palliat Med.

[15] Bravo G, Dubois MF, Wagneur B (2008). Assessing the effectiveness of interventions to promote advance directives among older adults: a systematic review and multi-level analysis. Soc Sci Med.

[16] Cane J, O'Connor D, Michie S (2012). Validation of the theoretical domains framework for use in behaviour change and implementation research. Implement Sci.

[17] Michie S, Johnston M, Abraham C, Lawton R, Parker D, Walker A (2005). Making psychological theory useful for implementing evidence based practice: a consensus approach. Qual Saf Health Care.

[18] Atkins L, Francis J, Islam R, O'Connor D, Patey A, Ivers N, Foy R, Duncan EM, Colquhoun H, Grimshaw JM (2017). A guide to using the theoretical domains framework of behaviour change to investigate implementation problems. Implement Sci.

[19] Just ask: A Conversation guide for Goals of Care Discussions. In . 2014.

[20] ADVANCE CARE PLANNING WORKBOOKS AND QUICK GUIDES [http://www.advancecareplanning.ca/resource/acp-workbook/]. Accessed in 2017.

[21] O’Brien BC, Harris IB, Beckman TJ, Reed DA, Cook DA (2014). Standards for reporting qualitative research: a synthesis of recommendations. Acad Med.

[22] Downar J, Goldman R, Pinto R, Englesakis M, Adhikari NK (2017). The "surprise question" for predicting death in seriously ill patients: a systematic review and meta-analysis. CMAJ.

[23] Michie S, van Stralen MM, West R (2011). The behaviour change wheel: a new method for characterising and designing behaviour change interventions. Implement Sci.

[24] Lund S, Richardson A, May C (2015). Barriers to advance care planning at the end of life: an explanatory systematic review of implementation studies. PLoS One.

[25] Durkin S, Brennan E, Wakefield M (2012). Mass media campaigns to promote smoking cessation among adults: an integrative review. Tob Control.

[26] Shirado A, Morita T, Akazawa T, Miyashita M, Sato K, Tsuneto S, Shima Y (2013). Both maintaining hope and preparing for death: effects of physicians' and nurses' behaviors from bereaved family members' perspectives. J Pain Symptom Manag.

[27] Bluhm M, Connell CM, De Vries RG, Janz NK, Bickel KE, Silveira MJ (2016). Paradox of prescribing late chemotherapy: oncologists explain. J Oncol Pract.

[28] Vehling S, Kamphausen A, Oechsle K, Hroch S, Bokemeyer C, Mehnert A (2015). The preference to discuss expected survival is associated with loss of meaning and purpose in terminally ill Cancer patients. J Palliat Med.

[29] Peter E, Mohammed S, Simmonds A (2015). Sustaining hope as a moral competency in the context of aggressive care. Nurs Ethics.

[30] Benzein E, Norberg A, Saveman BI (2001). The meaning of the lived experience of hope in patients with cancer in palliative home care. Palliat Med.

[31] Malcolm HA (2005). Does privacy matter? Former patients discuss their perceptions of privacy in shared hospital rooms. Nurs Ethics.

[32] van de Glind I, de Roode S, Goossensen A (2007). Do patients in hospitals benefit from single rooms? A literature review. Health Policy.

[33] Back AL, Arnold RM, Baile WF, Fryer-Edwards KA, Alexander SC, Barley GE, Gooley TA, Tulsky JA (2007). Efficacy of communication skills training for giving bad news and discussing transitions to palliative care. Arch Intern Med.

[34] Moore PM, Rivera Mercado S, Grez Artigues M, Lawrie TA. Communication skills training for healthcare professionals working with people who have cancer. Cochrane Database Syst Rev. 2013;(3):Cd003751. 10.1002/14651858.CD003751.pub3. 10.1002/14651858.CD003751.pub3 PMC6457800 23543521

[35] Curtis JR, Back AL, Ford DW, Downey L, Shannon SE, Doorenbos AZ, Kross EK, Reinke LF, Feemster LC, Edlund B (2013). Effect of communication skills training for residents and nurse practitioners on quality of communication with patients with serious illness: a randomized trial. Jama.

[36] De Vleminck A, Houttekier D, Pardon K, Deschepper R, Van Audenhove C, Vander Stichele R, Deliens L (2013). Barriers and facilitators for general practitioners to engage in advance care planning: a systematic review. Scand J Prim Health Care.

[37] Slort W, Schweitzer BP, Blankenstein AH, Abarshi EA, Riphagen II, Echteld MA, Aaronson NK, van der Horst H, Deliens L (2011). Perceived barriers and facilitators for general practitioner-patient communication in palliative care: a systematic review. Palliat Med.

[38] Beckman HB, Wendland M, Mooney C, Krasner MS, Quill TE, Suchman AL, Epstein RM (2012). The impact of a program in mindful communication on primary care physicians. Acad Med.

[39] Morton K, Beauchamp M, Prothero A, Joyce L, Saunders L, Spencer-Bowdage S, Dancy B, Pedlar C (2015). The effectiveness of motivational interviewing for health behaviour change in primary care settings: a systematic review. Health Psychol Rev.

