# Supplementary material for: Advance care planning after hospital discharge: qualitative analysis of facilitators and barriers from patient interviews
Source: BMC Palliat Care. 2018 Dec 5;17:127. doi: 10.1186/s12904-018-0379-0 (PMC6282276; doi:10.1186/s12904-018-0379-0)
Supplement: Supplementary file 1 — SRQR standards for Reporting Qualitative Research Checklist. Checklist for all elements that are suggested to be reported for qualitative research. (PDF 263 kb) [file 12904_2018_379_MOESM1_ESM.pdf]

Table 1

Standards for Reporting Qualitative Research (SRQR)[a]

No.	Topic		Item
	**Title and abstract**		
S1	Title	Yes P1	Concise description of the nature and topic of the study Identifying the study as qualitative or indicating the approach (e.g., ethnography, grounded theory) or data collection methods (e.g., interview, focus group) is recommended
S2	Abstract	Yes Structured	Summary of key elements of the study using the abstract format of the intended publication; typically includes background, purpose, methods, results, and conclusions
	**Introduction**		
S3	Problem formulation	Yes	Description and significance of the problem/phenomenon studied; review of relevant theory and empirical work; problem statement
S4	Purpose or research question	Yes	Purpose of the study and specific objectives or questions
	**Methods**		
S5	Qualitative approach and research paradigm	Yes Methods P 6+7	Qualitative approach (e.g., ethnography, grounded theory, case study, phenomenology, narrative research) and guiding theory if appropriate; identifying the research paradigm (e.g., postpositivist, constructivist/ interpretivist) is also recommended; rationale[b]
S6	Researcher characteristics and reflexivity	Yes P4+5	Researchers' characteristics that may influence the research, including personal attributes, qualifications/experience, relationship with participants, assumptions, and/or presuppositions; potential or actual interaction between researchers' characteristics and the research questions, approach, methods, results, and/or transferability
S7	Context	Yes P4	Setting/site and salient contextual factors; rationale[b]
S8	Sampling strategy	Yes P5	How and why research participants, documents, or events were selected; criteria for deciding when no further sampling was necessary (e.g., sampling saturation); rationale[b]
S9	Ethical issues pertaining to human subjects	Yes P4	Documentation of approval by an appropriate ethics review board and participant consent, or explanation for lack thereof; other confidentiality and data security issues
S10	Data collection methods	Yes Yes P 6	Types of data collected; details of data collection procedures including (as appropriate) start and stop dates of data collection and analysis, iterative process, triangulation of sources/methods, and modification of procedures in response to evolving study findings; rationale[b]
S11	Data collection instruments and technologies	Yes P6	Description of instruments (e.g., interview guides, questionnaires) and devices (e.g., audio recorders) used for data collection; if/how the instrument(s) changed over the course of the study
S12	Units of study	Yes P 5	Number and relevant characteristics of participants, documents, or events included in the study; level of participation (could be reported in results)
S13	Data processing	Yes P6	Methods for processing data prior to and during analysis, including transcription, data entry, data management and security, verification of data integrity, data coding, and anonymization/deidentification of excerpts
S14	Data analysis	Yes P6	Process by which inferences, themes, etc., were identified and developed, including the researchers involved in data analysis; usually references a specific paradigm or approach; rationale [b]
S15	Techniques to enhance trustworthiness		Techniques to enhance trustworthiness and credibility of data analysis (e.g., member checking, audit trail, triangulation); rationale[b]
	**Results/findings**		
S16	Synthesis and interpretation	P 7-11	Main findings (e.g., interpretations, inferences, and themes); might include development of a theory or model, or integration with prior research or theory
S17	Links to empirical data	P 11 - 13	Evidence (e.g., quotes, field notes, text excerpts, photographs) to substantiate analytic findings
	**Discussion**		
S18	Integration with prior work, implications, transferability, and contribution(s) to the field	P 11 -13	Short summary of main findings; explanation of how findings and conclusions connect to, support, elaborate on, or challenge conclusions of earlier scholarship; discussion of scope of application/ generalizability; identification of unique contribution(s) to scholarship in a discipline or field
S19	Limitations	P 13	Trustworthiness and limitations of findings

(*Table continues*)

Table 1

(Continued)

No.	Topic	Item
	**Other**	
S20	Conflicts of interest Title page	Potential sources of influence or perceived influence on study conduct and conclusions; how these were managed
S21	Funding N/A	Sources of funding and other support; role of funders in data collection, interpretation, and reporting

[a]The authors created the SRQR by searching the literature to identify guidelines, reporting standards, and critical appraisal criteria for qualitative research; reviewing the reference lists of retrieved sources; and contacting experts to gain feedback. The SRQR aims to improve the transparency of all aspects of qualitative research by providing clear standards for reporting qualitative research.

[b]The rationale should briefly discuss the justification for choosing that theory, approach, method, or technique rather than other options available, the assumptions and limitations implicit in those choices, and how those choices influence study conclusions and transferability. As appropriate, the rationale for several items might be discussed together.

should provide evidence (e.g., examples, quotes, or text excerpts) to substantiate the main analytic findings.[20,29]

Discussion. The discussion of qualitative results will generally include connections to existing literature and/or theoretical or conceptual frameworks, the scope and boundaries of the results (transferability), and study limitations.[10–12,28] In some qualitative traditions, the results and discussion may not have distinct boundaries; we recommend that authors include the substance of each item regardless of the section in which it appears.

Discussion

The purpose of the SRQR is to improve the quality of reporting of qualitative research studies. We hope that these 21 recommended reporting standards will assist authors during manuscript preparation, editors and reviewers in evaluating a manuscript for potential publication, and readers when critically appraising, applying, and synthesizing study findings. As with other reporting guidelines,[35–37] we anticipate that the SRQR will evolve as it is applied and evaluated in practice. We welcome suggestions for refinement.

Qualitative studies explore "how?" and "why?" questions related to social or human problems or phenomena.[10,38] Purposes of qualitative studies include understanding meaning from participants' perspectives (How do they interpret or make sense of an event, situation, or action?); understanding the nature and influence of the context surrounding events or actions; generating theories about new or poorly understood events, situations, or actions; and understanding the processes that led to a desired (or undesired) outcome.[38] Many different approaches (e.g., ethnography, phenomenology, discourse analysis, case study, grounded theory) and methodologies (e.g., interviews, focus groups, observation, analysis of documents) may be used in qualitative research, each with its own assumptions and traditions.[1,2] A strength of many qualitative approaches and methodologies is the opportunity for flexibility and adaptability throughout the data collection and analysis process. We endeavored to maintain that flexibility by intentionally defining items to avoid favoring one approach or method over others. As such, we trust that the SRQR will support all approaches and methods of qualitative research by making reports more explicit and transparent, while still allowing investigators the flexibility to use the study design and reporting format most appropriate to their study. It may be helpful, in the future, to develop approach-specific extensions of the SRQR, as has been done for guidelines in quantitative research (e.g., the CONSORT extensions).[37]

Limitations, strengths, and boundaries

We deliberately avoided recommendations that define methodological rigor, and therefore it would be inappropriate to use the SRQR to judge the quality of research methods and findings. Many of the original sources from which we derived the SRQR were intended as criteria for methodological rigor or critical appraisal rather than reporting; for these, we inferred the information that would be needed to evaluate the criterion. Occasionally, we found conflicting recommendations in the literature (e.g., recommending specific techniques such as multiple coders or member checking to demonstrate trustworthiness); we resolved these conflicting recommendations through selection of the most frequent recommendations and by consensus among ourselves.

Some qualitative researchers have described the limitations of checklists as a means to improve methodological rigor.[13] We nonetheless believe that a checklist for reporting standards will help to enhance the transparency of qualitative research studies and thereby advance the field.[29,39]

Strengths of this work include the grounding in previously published criteria, the diversity of experience and perspectives among us, and critical review by experts in three countries.

Implications and application

Similar to other reporting guidelines,[35–37] the SRQR may be viewed as a starting point for defining reporting standards in qualitative research. Although our personal experience lies in health professions education, the SRQR is based on sources originating in diverse health care and non-health-care fields. We intentionally crafted the SRQR to include various paradigms, approaches, and methodologies used in qualitative research. The elaborations offered in
