# Supplementary material for: Advance care planning after hospital discharge: qualitative analysis of facilitators and barriers from patient interviews
Source: BMC Palliat Care. 2018 Dec 5;17:127. doi: 10.1186/s12904-018-0379-0 (PMC6282276; doi:10.1186/s12904-018-0379-0)
Supplement: Supplementary file 2 — Semi Structured Interview Guide. The interview guide used for semi-structured interviews of patients (DOCX 17 kb) [file 12904_2018_379_MOESM2_ESM.docx]

**Appendix 1: Semi structured patient interview**

**All answers are confidential and will not be shown to doctors or other health care professionals who are responsible for your care. There are no right or wrong answers. Completely honest answers are most helpful!

1. During your hospital admission approximately 4 weeks ago, your doctor talked with you about advance care planning and we gave you some resources to help you in this process. Do you recall this discussion?

2) Can you tell me about the discussion you had with your doctor in hospital?

- *Explore the patient’s memory of the conversation*

Follow up questions:

What was good or helpful about this conversation?

What was difficult or frustrating about this conversation?

3) Did the doctor talk with you about prognosis and what changes to expect in your health over the next months-years? If so how did you feel about this?

Was any of this information surprising?

4) Did the discussion in hospital with your doctor encourage you to make an advance care plan or update your advance care plan? Why or why not?

- *Explore elements of the in hospital conversation that made the patient feel ACP was important for them or why they do not feel it is important for them.*

-What if anything could have been better about this conversation?

5) Have you met with your family doctor since being discharged to discuss advance care planning? If No, why? If Yes, can you tell me about the meeting?

*-Explore how this meeting was helpful to the patient.*

-Did this meeting help you create and Advance care plan?

6)* Do you have an advance directive or living will or some other written document describing the medical treatments they would want (or not want) in the event that you are unable to communicate for yourself as a result of a life threatening health problem? (Yes / No)

-*If No:* Do you want help to create an advanced care plan?

-*If Yes:* Did you create or update this document since being discharged from hospital?

-*If created or updated*: Did you use the advance care planning workbook we gave you on discharge to do this?

*If no* why not?

*If yes* Can you tell me about completing the workbook

What was difficult about it?

What was easy about it?

7)* Have you formally designated someone you trust (e.g. Power of Attorney for Health) to represent your wishes concerning medical treatment decisions in the event that you are not able to do so? (Yes / No)

-*If Yes:* Did you designate this person since being discharged from hospital?

* Adapted from previously validated survey: Heyland et al., J Palliative Care Med 2012, 2:5

** Taken Directly from the CANHELP Survey (http://thecarenet.ca/187-canhelp)
